# Annotations

**Published:** 1911-07-15

**Authors:** 


### Annotations.

### Medical Pensions in Ireland.

There are two very illuminating reports in the current issue of the *Journal of the Irish Medical Association* which show the kind of treatment with which long and faithful service to the public is rewarded by Boards of Guardians in the Sister Isle. The first deals with the pension awarded by the Swinford Guardians to Dr. O'Grady, for more than forty years medical officer to the workhouse, medical officer of health, and consulting sanitary officer of the entire area of the Swinford Union. The total maximum allowance which Dr. O'Grady is entitled to ask is £207 and a few shillings per annum; the award of the Guardians was £80. We are glad to note that the Local Government Board have written expressing "disappointment" at this beggarly pension for one whose valuable services are acknowledged by a resolution passed by the Guardians themselves. In fact, the Board invites the Guardians to reconsider the decision in language which will doubtless lead to some more satisfactory arrangement. Hard upon this case, the Tobercurry Guardians were called upon to fix the pension of their workhouse medical officer, who had only thirty-two years of devoted service to his credit. The maximum in this case was £226, and the first resolution proposed that this sum should be awarded. Then one member quoted the £80 deemed sufficient at Swinford, and moved that the grant be £100. The next suggestion was £177, followed by another of £130, and a fifth of £150: these on the ground that most of the grant would come out of public instead of local funds! The Chairman favoured the third suggestion (£177), but in the end the original resolution was carried, and thus Tobercurry has escaped the Government reproof which justly reached Swinford. But the incidents throw a somewhat lurid light on the prospects of medical officers in Ireland, and on the degrading elements necessarily introduced when party politicians are allowed to meddle thus in matters with which they should by right have no concern whatever.

### The Zebrules at the Zoo.

A writer in the *Times*, noticing the recent birth of two wild ass-zebra hybrids at the Zoological Gardens, opines that although hybrids in the case of the equidæ are usually infertile, like the ordinary mule, yet in the above instance there may be a prospect of progeny, since donkeys and zebras are undoubtedly more closely allied than are either of them to horses. As a matter of fact, in Mr. W. B. Tegetmeier's "Horses, Asses, Zebras, Mules, and Mule-breeding," one case is quoted of a donkey-zebra hybrid producing a foal with a pony. If the report be correct, as from its citation by an authority like Mr. Tegetmeier one would undoubtedly expect it to be, then there would be very good prospect of one of the present hybrids proving fertile with either ass or zebra. We note that the zebras used in the experiments at the Zoological Gardens were the one a mountain zebra, and the other Chapman's variety. As these are respectively an asinine and an equine type of this animal, perhaps the likelier hybrid to mate with horses successfully would be the offspring of the Chapman's zebra. Some blood work on these little creatures when they are old enough would be interesting, for, as readers familiar with the literature of the precipitin test will recall, the blood of horses and of zebras shows affinity. This test, by the way, has just for the first time in this country served the ends of justice, as noticed by the presiding judge. It is another sign of the increasing importance to the community of the medical profession, a fact the manifold bearings of which we have repeatedly insisted upon.

### The Sherborne School Case.

It is a little difficult to understand how Mr. Lindner's advisers can have allowed him to go into court on the flimsy case which was presented in respect of his son's unfortunate illness last week. With every sympathy for the boy's cruel fate, it is impossible not to agree with the remark of the Lord Chief Justice that he could not have allowed any other verdict to stand. The sequence of events as elicited in evidence is fairly simple. Mr. Lindner, junior, was known to be a somewhat delicate boy, though free from any actual ill-health, and was excused from compulsory games on that account. He was destined for a career as a violinist. Before the end of the Michaelmas term last year he had a superficial whitlow on the left thumb, which he reported to the matron. The latter passed him on to the school doctor, who incised the whitlow without anæsthesia. When he went home for the Christmas holidays he had some cough, and he was cautioned to see his own doctor at home. Later still he developed an axillary abscess on the *right* side, and eventually his arm had to be amputated for some septicæmic condition, after which he recovered. To plead that a superficial whitlow on the left hand, duly incised and fomented, could reasonably be held as neglected because later on trouble develops on the right side, is a chain of reasoning so defective that it is not astonishing the expert witnesses called were able to upset it. Curiously enough, the school doctor was not made a party to the action, though he had an opportunity of clearing his professional character in the witness-box. In congratulating him and the other defendants on the upshot of the case, it is worth noting how great a responsibility school doctors incur in treating the minor ailments of that happy-go-lucky and uncomplaining person, the English public schoolboy. Had the axillary infection been on the left side instead of the right, there might have been much greater difficulty in persuading the jury to take the same view of the case.

